# Workshop on Risks Associated with Transmissible Spongiform Encephalopathies (TSEs)

**DOI:** 10.3201/eid0501.990132

**Published:** 1999


ing high quality scientific evidence that will define the true role of infections and inflammation in atherosclerotic disease and the appropriateness of antimicrobial therapy.

For more information on the conference, contact Siobhán O'Connor, Centers for Disease Control and Prevention, 1600 Clifton Rd., NE, Mailstop C12, Atlanta, GA 30333, USA; tel: 404-639-1454; fax: 404-639-3039; e-mail: sbo5@cdc.gov.

### Workshop on Risks Associated with Transmissible Spongiform Encephalopathies (TSEs)

A workshop on the evaluation of risks posed by TSEs was held June 8-9, 1998, at the University of Maryland College Park, under the auspices of the Joint Institute for Food Safety and Applied Nutrition (JIFSAN), a cooperative venture of the University of Maryland and the U.S. Food and Drug Administration. The workshop, attended by 300 representatives from 17 countries, evolved in response to the need voiced by governmental agencies, industry groups, international organizations, and academic experts to evaluate TSE risks related to source materials, processing, and end products for animal and human use. Support for the workshop came from government agencies, industry groups, international organizations, and JIFSAN.

One goal of the workshop was to develop a framework of guiding principles for evaluating TSE risks. An initial draft outline of critical elements applicable to evaluations of TSE risk was presented. The next steps are to provide definitions for the critical elements and to develop an annotated outline that explains the relevance of each key element. A set of information tools (under development) to facilitate risk evaluation and access to TSE knowledge was demonstrated at the workshop.

The workshop 1) identified research needs and reviewed risks associated with sources of raw materials (material collection and procurement are primary control points for ensuring low risk of TSE transmission); 2) focused on inactivation schemes for TSE agents, which may be the most reliable way of reducing the level of TSE agent; 3) examined use of several categories of end products: foods, pharmaceuticals, cosmetics, blood and blood products, biologics, and tissues of animal and human origin; and 4) proposed strategies for TSE risk evaluation.

Representatives of governments, private organizations, and industry groups presented their approaches to the evaluation of TSE risks and found common themes: 1) zero risk does not exist; 2) decisions concerning public health issues must often be made in the absence of complete information; 3) risk analysis may be useful in understanding the key elements of a problem or situation; 4) the risk evaluation process must be responsive to rapidly changing information and new scientific data; 5) the risk evaluation process should be transparent, involving all partners (general public, regulated industry, government); 6) disagreements still exist concerning the appropriateness of models, assumptions, and methods; and 7) although both qualitative and quantitative approaches to TSE risk evaluation have merit, no single approach is applicable to all situations.

Workshop documents, including illustrated transcripts of the presentations, a draft outline of critical elements in TSE risk evaluations, observations on human and animal issues, and workshop overviews, are available at http://www.life.umd.edu/jifsan/tse.html).

### USDA Report on Potential for International Travelers to Transmit Foreign Animal Diseases to U.S. Livestock or Poultry

The U.S. Department of Agriculture has published a new report—The Potential for International Travelers to Transmit Foreign Animal Diseases to U.S. Livestock or Poultry—which explores the issue of animal disease transmission by human travelers. Millions of people travel internationally each year, for both pleasure and business, and thus may be at risk for being infected by animals and causing infections in animals.

The report highlights and summarizes what is currently known about the potential for human infection and human-to-animal transmission of foreign animal diseases. It focuses on the International Office of Epizootics List A diseases. These transmissible animal diseases, which may spread rapidly and have serious

A workshop on the evaluation of risks posed by TSEs was held June 8-9, 1998, at the University of Maryland College Park, under the auspices of the Joint Institute for Food Safety and Applied Nutrition (JIFSAN), a cooperative venture of the University of Maryland and the U.S. Food and Drug Administration. The workshop, attended by 300 representatives from 17 countries, evolved in response to the need voiced by governmental agencies, industry groups, international organizations, and academic experts to evaluate TSE risks related to source materials, processing, and end products for animal and human use. Support for the workshop came from government agencies, industry groups, international organizations, and JIFSAN.

One goal of the workshop was to develop a framework of guiding principles for evaluating TSE risks. An initial draft outline of critical elements applicable to evaluations of TSE risk was presented. The next steps are to provide definitions for the critical elements and to develop an annotated outline that explains the relevance of each key element. A set of information tools (under development) to facilitate risk evaluation and access to TSE knowledge was demonstrated at the workshop.

The workshop 1) identified research needs and reviewed risks associated with sources of raw materials (material collection and procurement are primary control points for ensuring low risk of TSE transmission); 2) focused on inactivation schemes for TSE agents, which may be the most reliable way of reducing the level of TSE agent; 3) examined use of several categories of end products: foods, pharmaceuticals, cosmetics, blood and blood products, biologics, and tissues of animal and human origin; and 4) proposed strategies for TSE risk evaluation.

Representatives of governments, private organizations, and industry groups presented their approaches to the evaluation of TSE risks and found common themes: 1) zero risk does not exist; 2) decisions concerning public health issues must often be made in the absence of complete information; 3) risk analysis may be useful in understanding the key elements of a problem or situation; 4) the risk evaluation process must be responsive to rapidly changing information and new scientific data; 5) the risk evaluation process should be transparent, involving all partners (general public, regulated industry, government); 6) disagreements still exist concerning the appropriateness of models, assumptions, and methods; and 7) although both qualitative and quantitative approaches to TSE risk evaluation have merit, no single approach is applicable to all situations.

Workshop documents, including illustrated transcripts of the presentations, a draft outline of critical elements in TSE risk evaluations, observations on human and animal issues, and workshop overviews, are available at http://www.life.umd.edu/jifsan/tse.html).

